# Development and psychometric evaluation of a patient-reported symptom index for patients with non-muscle invasive bladder cancer: the NMIBC-SI

**DOI:** 10.1186/s41687-025-00864-7

**Published:** 2025-03-27

**Authors:** Claudia Rutherford, Margaret-Ann Tait, Daniel S. J. Costa, Madeleine T. King, David P. Smith, Shomik Sengupta, Joseph Ischia, Andrew Mitterdorfer, Dickon Hayne, Roger Watson, Paul Anderson, Mark Frydenberg, Peter Gilling, Nicholas Buchan, Euan Green, Noel Clarke, Stephen A. Boorjian, Badrinath Konety, Jeffrey M. Holzbeierlein, Peter C. Black, Venu Chalasani, Jörg Henseler, Manish I. Patel, on behalf of the NMIBC-SI Study Group

**Affiliations:** 1 Faculty of Medicine and Health, Susan Wakil School of Nursing and Midwifery, University of Sydney, Sydney, Australia; 2 The Daffodil Centre, The University of Sydney, A Joint Venture with Cancer Council New South Wales, Sydney, NSW Australia; 3 Faculty of Science, School of Psychology, University of Sydney, Sydney, NSW Australia; 4 Pain Management Research Institute, Royal North Shore Hospital, St Leonards, NSW Australia; 5 Eastern Health Clinical School, Monash University, Melbourne, VIC Australia; 6 ANZUP Cancer Trials Group, Sydney, NSW Australia; 7 Department of Surgery, University of Melbourne, Austin Health, Heidelberg, VIC Australia; 8 Concord Repatriation General Hospital, Concord, NSW Australia; 9 UWA Medical School, University of Western Australia, Perth, WA Australia; 10 Mater Hospital, South Brisbane, QLD Australia; 11 Royal Melbourne Hospital, Parkville, VIC Australia; 12 Monash Medical Centre, Department of Surgery, Monash University, Melbourne, VIC Australia; 13 Tauranga Urology Research, Tauranga, New Zealand; 14 Canterbury Urology Research Trust, Forte Health, Christchurch, New Zealand; 15 The Christie and Salford Royal Hospitals, Manchester, UK; 16 Mayo Clinic, Rochester, MN USA; 17 University of Minnesota, Minneapolis, MN USA; 18 University of Kansas Health System, Kansas City, KS USA; 19 University of British Columbia, Vancouver, BC Canada; 20 University of Sydney, Northern Clinical School, Sydney, NSW Australia; 21 University of Twente, Enschede, The Netherlands; 22 Nova Information Management School, Universidade Nova de Lisboa, Lisbon, Portugal; 23 Sydney Medical School, Specialty of Surgery, University of Sydney, Sydney, NSW Australia; 24 Department of Urology, Westmead Hospital, Westmead, NSW Australia; 25 Sydney Quality of Life Office, Faculty of Medicine and Health, University of Sydney, Sydney, NSW Australia

**Keywords:** Early bladder cancer, Measurement properties, Non-muscle invasive bladder cancer, Patient-reported outcome, Patient-reported outcome measure, Symptom benefit, Symptom burden, Symptom index

## Abstract

**Background and objective:**

Non-muscle invasive bladder cancer (NMIBC) is a chronic condition requiring frequent follow-up with endoscopic examinations, tumour resections and intravesical treatments. In this clinical context, patient-reported outcomes (PROs) have enormous potential to inform treatment assessment and recommendations for NMIBC. We aimed to develop and evaluate a patient-reported NMIBC Symptom Index (NMIBC-SI) to facilitate clinical research and enhance care.

**Methods:**

NMIBC-SI items were developed based on existing literature and qualitative interviews with patients and clinicians, and evaluated in two field tests: item reduction, using NMIBC-SI data from 220 patients on active treatment from nine Australian centres; reliability and validity evaluation of item-reduced version using NMIBC-SI data from 232 patients from five countries.

**Results:**

NMIBC-SI assesses disease and treatment-related symptom burden and two treatment-specific side-effects (cystoscopy, intravesical BCG/Chemotherapy). Composite analysis supported a single composite model including core symptom and cystoscopy index items (Intravesical index items were not tested due to small sample). Test-retest reliability was strong (range 0.894–0.91). As expected, the NMIBC-SI was able to discriminate between no treatment and any treatment groups, and no treatment and chemo/BCG groups, providing evidence towards validity.

**Conclusions and clinical implications:**

NMIBC-SI assesses patients’ self-reported symptom burden and can be used to evaluate NMIBC treatments from the perspective of patients. The NMIBC-SI is acceptable to patients and has evidence for reliability and validity. Future validation work with patients with greater symptom burden is warranted.

**Supplementary Information:**

The online version contains supplementary material available at 10.1186/s41687-025-00864-7.

## Abstract (plain language summary)

Patients with NMIBC require long-term monitoring with regular endoscopic examinations and various treatments. This research describes the development of the NMIBC-SI, a patient-completed questionnaire that assesses the impact of those treatments on health outcomes important to patients. The NMIBC-SI can be used in research and in the clinic to improve communication and treatment decisions.

**Supplementary Information:**

The online version contains supplementary material available at 10.1186/s41687-025-00864-7.

## Introduction

Bladder cancer (BC) is the ninth most common cancer worldwide with an estimated 614,298 people diagnosed in 2022 [1]. Most cases (70–80%) diagnosed are non-muscle invasive bladder cancer (NMIBC) [2], with 60–70% recurring, and 5-year progression to muscle invasion ranging from 1% for low risk to 21% for high risk [3], with a 40% probability of progression in a recently identified *very high* risk group [4]. For NMIBC, five year survival is > 80% [5], yielding prevalence 10 times greater than incidence rates [6]. Treatment for NMIBC involves endoscopic resection of bladder tumours, usually followed by intravesical chemotherapy or immunotherapy in recurrent and/or high-risk cases. Intensive follow-up is mandatory, with repeated endoscopic examinations ± bladder biopsy/tumour resection initially 3-monthly, decreasing according to risk and radiological imaging. Intravesical treatments are used when appropriate. Adjuvant therapies may reduce recurrence, but have sequelae such as flu-like symptoms, urinary frequency, urgency, fatigue, and dysuria. Follow-up examinations are recommended to detect and treat recurrences. In this context, patient-reported outcomes (PROs) including symptom burden, physical function, and health-related quality of life (HRQL) are important issues for patients and managing clinicians.

A PRO is a report coming directly from patients, without interpretation by another, about how they feel in relation to a health condition and its therapy [7]. The role of PRO measures (PROMs) is important in clinical trials that evaluate the effectiveness of treatment because they incorporate the patient’s perspective. In oncology, PROs are useful for improving service provision and informing treatment decisions [8]. PROs are particularly important where survival outcomes may not be the most relevant treatment outcome, particularly in chronic conditions such as NMIBC. They also offer a patient-centred measure of treatment impact, burden on patients and presence of symptoms or side-effects not assessable with clinical tests [9]. Due to the combination of these factors, there is enormous potential for PROs to inform treatment assessment, patient information and recommendations for NMIBC management.

Despite a large number of RCTs conducted (~ 200 since 1970) evaluating effectiveness of NMIBC treatments, many focused on assessing adverse events, with few assessing PROs in the short and long-term [10]. Where PROs were assessed, the evidence is limited by small sample sizes, lack of comparison groups, poor adjustment for baseline function, and failure to distinguish between patients with varying degrees of risk and between treatments in the analysis [11]. Consequently, key evidence on the impact of contemporary therapies for NMIBC on patient’s HRQL is lacking [10]. 

NMIBC generally has good clinical outcomes with available treatment options. Hence choice of treatment should consider additional aspects such as trade-offs in certain PROs. Our earlier work developed an empirically derived conceptual framework of PROs important to people with NMIBC, including proximal effects that can occur directly as a consequence of the NMIBC and/or treatment for the disease (e.g. blood in urine, urinary frequency) and their consequential effects on a person’s ability to function and their overall well-being (i.e. distal effects) [12]. This framework includes six domains covering symptoms and treatment side-effects, five functioning domains, and seven experience of care domains, guiding the design of a comprehensive PRO assessment plan for clinical practice in NMIBC and future clinical trials of treatments for NMIBC.

We conducted a systematic review of PROMs available to assess PROs identified in our conceptual framework, evaluating optimality (i.e. efficient and focused measurement) of existing PROMs as measures of symptom benefit for use as clinical trial endpoints in contemporary treatment for NMIBC [11]. We found that some PROs important in NMIBC were inadequately covered by generic and cancer-specific PROMs (e.g. skin problems, severe systemic side-effects of treatment such as joint pain and stiffness, physical function impairment due to restricted movement, psychological distress) and several PROMs used in NMIBC studies contained some content irrelevant to people undergoing contemporary treatment for NMIBC (e.g. appetite, nausea, vomiting) [11]. The European Organisation for Research and Treatment of Cancer (EORTC) have developed a suite of PROMs designed to assess HRQL in cancer clinical trials. The Quality of Life Questionnaire (QLQ-C30) is a core 30-item PROM designed to measure HRQL in all cancer patients [13]. Disease and treatment-specific modules complement the core measure, improving the sensitivity and specificity of HRQL assessments in specific groups of patients (for example, the QLQ-NMIBC24 – a disease-specific module assessing HRQL issues affecting patients with NMIBC) [14]. The EORTC QLQ-C30 plus the EORTC QLQ-NMIBC24 module was best aligned with our NMIBC-specific conceptual model [12], but failed to represent some PROs important to NMIBC patients (e.g. skin problems, joint pain/stiffness).

The EORTC PROMs were developed to enable a comprehensive HRQL assessment, assessing a range of issues including aspects of functioning, side-effects of cancer treatment, and symptoms of specific cancers. Symptom indexes, on the other hand, are PROMs which provide a more focused approach, typically including only relevant symptoms/side-effects summed into a single index. This offers an alternative measurement approach to the EORTC’s more comprehensive and modular approach to the assessment of HRQL. A brief focussed symptom measure intended to comprehensively assess direct symptoms and side-effects of contemporary treatments for NMIBC would enable accurate, robust, and clinically relevant assessment of differences in PROs among current and emerging therapies for NMIBC.

Extensive work with patients was conducted previously to develop and pre-test a draft NMIBC-specific symptom index (NMIBC-SI) [12, 15, 16]. First, an exhaustive list of clinically relevant issues was generated from three sources: (1) a systemic review and narrative analysis of the NMIBC-PRO literature [12]; (2) in-depth qualitative interviews with 26 NMIBC patients that explored patients’ experience of receiving treatment; and (3) in-depth qualitative interviews with 20 treating clinicians (specialist nurses and urologists) [12]. The list of issues was framed into questions (items) and constructed into the draft NMIBC-SI. This version was pre-tested with 15 patients and clinicians for content clarity and comprehensiveness, and appropriateness of the NMIBC-SI’s time frame, question stem, and response options [16]. 

This study aimed to:

1. Undertake a preliminary evaluation of the draft NMIBC-SI by examining the legitimacy of summing items into an index and identify items with poor psychometric performance for possible elimination (field test 1).
2. Psychometrically evaluate reliability, validity, and clinical utility of the final NMIBC-SI (field test 2).

## Methods

This multi-centre study included two field tests. Field test 1 was a national cross-sectional study involving NMIBC patients aged ≥ 18, recruited during active treatment or within one week after final treatment. Eligible patients were recruited from nine Australian sites by treating urologists and urology nurses between February 2017 and March 2018. Data collected included patient demographics, clinical features of the tumour, risk category [17], treatment type, comorbidity, and the draft NMIBC-SI.

Field test 2 was a prospective longitudinal study involving patients aged ≥ 18, newly diagnosed with NMIBC after imaging or flexible cystoscopy but before endoscopic resection. Eligible patients were recruited from nine Australian, two New Zealand, three United States, one United Kingdom, and one Canadian, sites by treating urologists and urology nurses between July 2018 and July 2020. Data collected included patient demographics, clinical features of the tumour, risk category [17], treatment type, comorbidity, the NMIBC-SI, and HRQL assessed with EORTC QLQ-C30 [13] and QLQ-NMIBC24 [14] self-completed in English at baseline and four follow-up times (Table 1). Detailed methods are reported in the study protocol [15]. 

**Table 1:** PRO assessment schedule

Risk Group	Time 1 (t1) Assessed within 3 months before tumour resection	Time 2 (t2) Assessed within 4 to 10 days after tumour resection	Time 3 (t3) Assessed within 1 month after the end of induction intravesical therapy	Time 4 (t4) Assessed 3–7 days after Time 3	Time 5 (t5) Assessed within 1 month prior to 1-year cystoscopy (or at early cessation due to adverse events)
High	t1	t2	t3	t4	t5
Intermediate	t1	t2	t3	t4	t5
Low	t1	t2	t3*	t4	t5
~n	250	250	250	75	250

* For the low risk group, t3 was 8 weeks after resection

The pre-tumour resection assessment (t1) was at diagnosis and considered the baseline assessment. Patients were not assumed to have had any treatment-related problems at this point. Time-points 2, 3 and 5 were intended to capture short, intermediate, and long-term levels of treatment-related problems. Time-point 4 was between 3–7 days after t3 to enable test-retest analysis

### Analysis

#### Field test 1

Our conceptual framework comprised physical symptoms caused by NMIBC or treatment, physical movement, psychological symptoms, sexual function, treatment burden, cystoscopy side-effects, and Bacillus Calmette-Guerin (BCG)/chemotherapy side-effects [12]. The symptom index included only the physical symptoms (Core index) and side-effects of cystoscopy and intravesical therapy (BCG/chemotherapy), assessed across 88-items.

We adopted a clinimetric rather than psychometric approach to analysis [18], as our goal was to identify key symptoms for inclusion, regardless of their co-occurrence and/or correlations with other items. Items were selected for their clinical relevance and combined into clinically-meaningful groups [19], rather than positing the existence of a latent variable and selecting a sample of interchangeable items to represent such a variable, as in the reflective approach [20–22]. Items were composite (formative) indicators [20], which need not be strongly correlated with each other to form a ‘composite’ [20] or ‘index’ [23], thus rendering factor analysis, item response theory, and measures of internal consistency inappropriate [22, 23]. Instead, we flagged items for possible exclusion based on the following:

- Low prevalence - frequency of each item response option (floor/ceiling effects), mean, and standard deviation was calculated. Items with marked floor/ceiling effects were considered for exclusion;
- High missing data - Items with > 20% missing data were considered for exclusion. The denominator was those patients to whom the items were applicable (e.g. having BCG);
- Redundancy – items conceptually and/or functionally similar and highly correlated (Pearson correlation ≥ 0.50) with an already included item (to avoid double-counting of essentially the same symptom). We calculated correlation coefficients for each pair of items and examined all correlations in descending order of magnitude. Pairs that correlated > 0.5 were flagged for further scrutiny and decisions about retaining/removing items were made by an expert group of nine urologists and the PRO methodologists on the team. The item considered to be phrased most clearly was retained, the other considered redundant and removed;
- Patient-reported experiences (PREs) of care (e.g. “treatment inconvenience”) rather than symptoms of disease or treatment. Questionnaires assessing PREs of care are available [24, 25] and could be used in studies with PRE endpoints;
- Too general (could have a range of causes; e.g. bodily pain) or not NMIBC-specific (e.g. cough).

Nine urologists reviewed all items and results from field test 1 analysis and provided input to item inclusion/exclusion decisions. Clinicians selected items to retain from items that were highly correlated and perceived to be assessing the same concept (e.g. blood in urine retained, passing blood clots removed) and ensured clinically important items were not excluded due to low prevalence (e.g. fever), until consensus was reached by all nine urologists.

#### Field test 2

Descriptive statistics included participant demographics, clinical characteristics, and loss to follow-up at each time-point.

### Composite analysis

We used Confirmatory Composite Analysis (CCA) [26, 27] to examine the NMIBC-SI. To maximise data available for this analysis, we combined data from different time-points. For all analyses we used T3 if available. If T3 was missing for a participant we used their T4 data, if T4 was also missing we used their T5 data, and if T5 was also missing we used their T2 data. We first examined response frequencies for each item in the data set to identify skew and/or floor/ceiling effects.

CCA is described in detail elsewhere [26–28]. Briefly, whereas factor analysis evaluates items based on inter-item correlations (assuming a latent variable as a common underlying cause), *composites* are evaluated on the basis of their covariance with external criteria; thus, the focus in on the utility of a collection of items, not how strongly they are associated with one another. A composite variable is an exact linear combination of its indicators, such that the construct of interest is defined by its indicators. This approach is arguably better suited to the validation of symptom indexes than traditional approaches [28]. 

We tested three models (Table 2). Because CCA analyses uses complete cases, and only a small subset (*N* = 65) of the sample completed the Intravesical index, this index was excluded from CCA. CCA was conducted using the cSEM package in R [29]. 

To determine acceptability of each index score we examined score distributions and floor/ceiling effects (Table 2).

Test-retest reliability of index scores was assessed with single measurement, random effects intra-class correlations (ICCs) in psych() package using data from T3 and 3–7 days after T3 (T4; Table 1); we anticipated scores to be relatively stable at these times (Table 2).

Clinical known-groups validity was compared for NMIBC-SI scores for patients by treatment groups. We hypothesised: at T3, patients experiencing any treatment (transurethral removal of bladder tumour (TURBT), chemotherapy, BCG) would have greater symptom burden than those experiencing no treatment; patients experiencing chemotherapy/BCG would have greater symptom burden than those experiencing no treatment; and those undergoing BCG would have greater symptom burden than those undergoing chemotherapy (Table 2).

**Table 2:** Psychometric tests and criteria to assess reliability and validity

Psychometric property	Definition/Test	Criteria for Acceptability
**Construct validity**	Composite Analysis tested 3 models: 1. One-composite model, comprising the 23 *core symptom index* items. We used *quality of life* as the criterion variable, which was modelled as a reflective latent variable with two EORTC QLQ-C30 global health and quality of life items as its indicators. 2. Two-composite model, comprising the *core index* composite and a *cystoscopy* composite (6 items). 3. One-composite model, where composite comprised all 29 core and cystoscopy items. In anticipation of multicollinearity between indicators within a domain, we used Mode A, which estimates weights using simple rather than multiple regression, i.e., it does not control for other indicators.	Models were evaluated using *R*^2^, comparative fit index (CFI), Tucker-Lewis index (TLI), root mean square error of approximation (RMSEA) and the standardised root mean residual (SRMR). Criteria for good fit were as follows: CFI > 0.9, TLI > 0.9, RMSEA < 0.08, SRMR < 0.05. We also examined the Akaike and Bayesian Information Criteria (AIC and BIC) to determine the best fitting model.
**Acceptability – Index-Level Performance**	For each index score, we examined score distributions to identify skew and floor/ceiling effects.	• normal distribution of endorsement frequencies across response categories (i.e. absence of skew, endorsement rates between 0.20 and 0.80) • floor/ceiling effects for Index scores < 10%
**Test-retest reliability**	The stability of a measuring instrument; assessed by administering the instrument to respondents on two different occasions and examining the correlation between test and retest scores.	ICCs for summary scores of ≥ 0.70 indicated significant correlations between scale scores at the two-time points.
**Clinical validity** (sensitivity to differences between patient groups)	The ability of a scale to differentiate known groups; assessed by comparing scores for subgroups who are expected to differ on the construct being measured.	Hypotheses tested using independent-samples *t*-tests with Welch’s adjustment for unequal variances, and standardised mean differences, defined as the mean difference divided by its standard deviation, [30] and 95% confidence intervals (CI).

## Results

### Field test 1

Field test 1 included 220 participants: 178 male, mean age 69.3, representing all risk groups (Table 3 and Table S1). Of 88 items, most participants (> 80%) did not report experiencing 21 items. Seven highly correlated items were excluded, seven excluded as > 50% of urologists rated them as not directly related to NMIBC treatment or considered too general (e.g. “have you had a cough?”), 30 physical, psychological and sexual function items were excluded because distal impacts of treatment on physical, psychological and sexual function are covered in existing PROMs developed for assessing these domains. This produced a 23-item NMIBC-SI assessing disease or treatment-related symptoms (NMIBC-Core, 23-items) and two treatment-specific optional indexes assessing cystoscopy-specific (NMIBC-Cyst, 6-items) or intravesical BCG/Chemotherapy-related side-effects (NMIBC-Intra, 10-items).

**Table 3:** Field test 1 participant demographic and clinical information (*n* = 220)

Characteristic	Total (*n* = 220)
**Age**	
Mean (Standard deviation)	69.3 (10.9)
Median Age in years (Range)	69 (33–90)
Interquartile range (IQR)	63, 77
Missing	19 (8.6%)
**Gender**, ***n (%)***	
Male	178 (81%)
Female	42 (19%)
**Education**, ***n (%)***	
Primary School	11 (5%)
High School	111 (50.5%)
Certificate or Diploma	63 (28.6%)
Degree or higher	31 (14.1%)
Missing	4 (1.8%)
**Setting**, ***n (%)***	
Public (8 sites)	178 (81%)
Private (2 sites)	42 (19%)
**Tumour progression risk classification**, ***n (%)***	
Low	61 (27.7%)
Intermediate	29 (13.2%)
High	112 (50.9%)
Missing	18 (8.2%)
**Grade of Tumour**, ***n (%)***	
PUNLMP	2 (1%)
Low	76 (34.5%)
High	121 (55%)
Missing	21 (9.5%)
**Stage of Tumour**, ***n (%)***	
CIS (carcinoma in situ)	33 (15%)
pTa	127 (57.7%)
pT1	38 (17.3%)
Missing	22 (10%)
**Treatments patients currently receiving**, *n (%)*(maybe more than one) *	
Cystoscopy exam with or without biopsy	124 (56.4%)
TURBT	61 (27.7%)
Single instillation Chemotherapy	5 (2.3%)
Induction instillation Chemotherapy	13 (5.9%)
Maintenance chemotherapy	1 (0.5%)
Induction instillation BCG	63 (28.6%)
Maintenance BCG	44 (20%)
Other	3 (1.4%)
Missing	18 (8.2%)
**Time since last cystoscopy**, ***n (%)***	
Assessed before or on the day of cystoscopy	71 (32.3%)
Assessed 1–21 days after cystoscopy	45 (20.5%)
Assessed more than 21 days after cystoscopy	84 (38.2%)
Missing	20 (9%)
**Time since last BCG or Chemo Treatment**, ***n (%)***	
Assessed < 1 month after BCG/Chemo	38 (17.3%)
Assessed > 1 month after BCG/Chemo	66 (30%)
Not receiving BCG/Chemo Treatment	98 (44.5%)
Missing	18 (8.2%)

PUNLMP papillary urothelial neoplasm of low malignant potential; TURBT transurethral resection of bladder tumour

* Values indicate that patients were receiving multiple treatments at the time of their assessment

### Field test 2

**Fig. 1:**
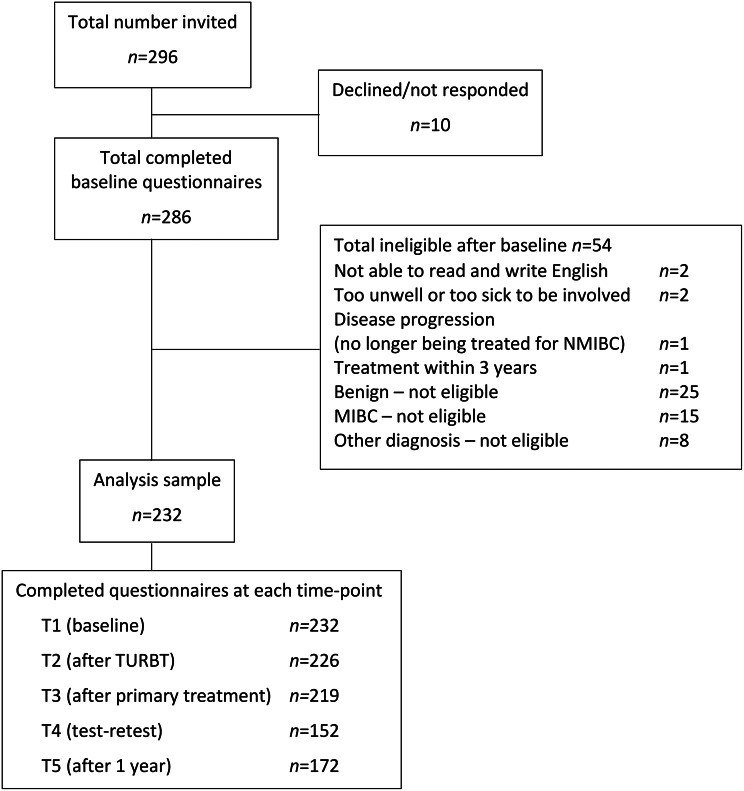
Flow of participants through field test 2

Field test 2 included 232 participants (Fig. 1): 176 males, mean age 68yrs, representing Low 53.5%, Intermediate 11.2% and High 25.4% risk groups [17] (Tables 4 and S2). Average NMIBC-SI completion time was 3 min, 41 sec.

**Table 4:** Field test 2 participant baseline demographic and clinical information (*n* = 232)

Characteristic	Total (%)
Median age (Range)	71 (32–90)
Mean age (SD)	68 (10.52)
Missing	4
Sex ratio (Male: Female)	176:56
Missing	0
**Participating countries**, ***n (%)***	
Australia	95 (40.9%)
New Zealand	56 (24.1%)
USA	42 (18.1%)
United Kingdom	21 (9.1%)
Canada	9 (3.9%)
Withdrawn	9 (3.9%)
**Education level**, ***n (%)***	
Primary school	15 (6.5%)
High school	100 (43.1%)
Technical College	48 (20.7%)
University or above	56 (24.1%)
Missing	13 (5.6%)
**Treatment at baseline**, ***n (%)***	
Flex Cystoscopy	118 (50.9%)
Cystoscopy Biopsy	18 (7.8%)
None	86 (37.1%)
Other	15 (6.5%)

### Composite analysis

**Fig. 2:**
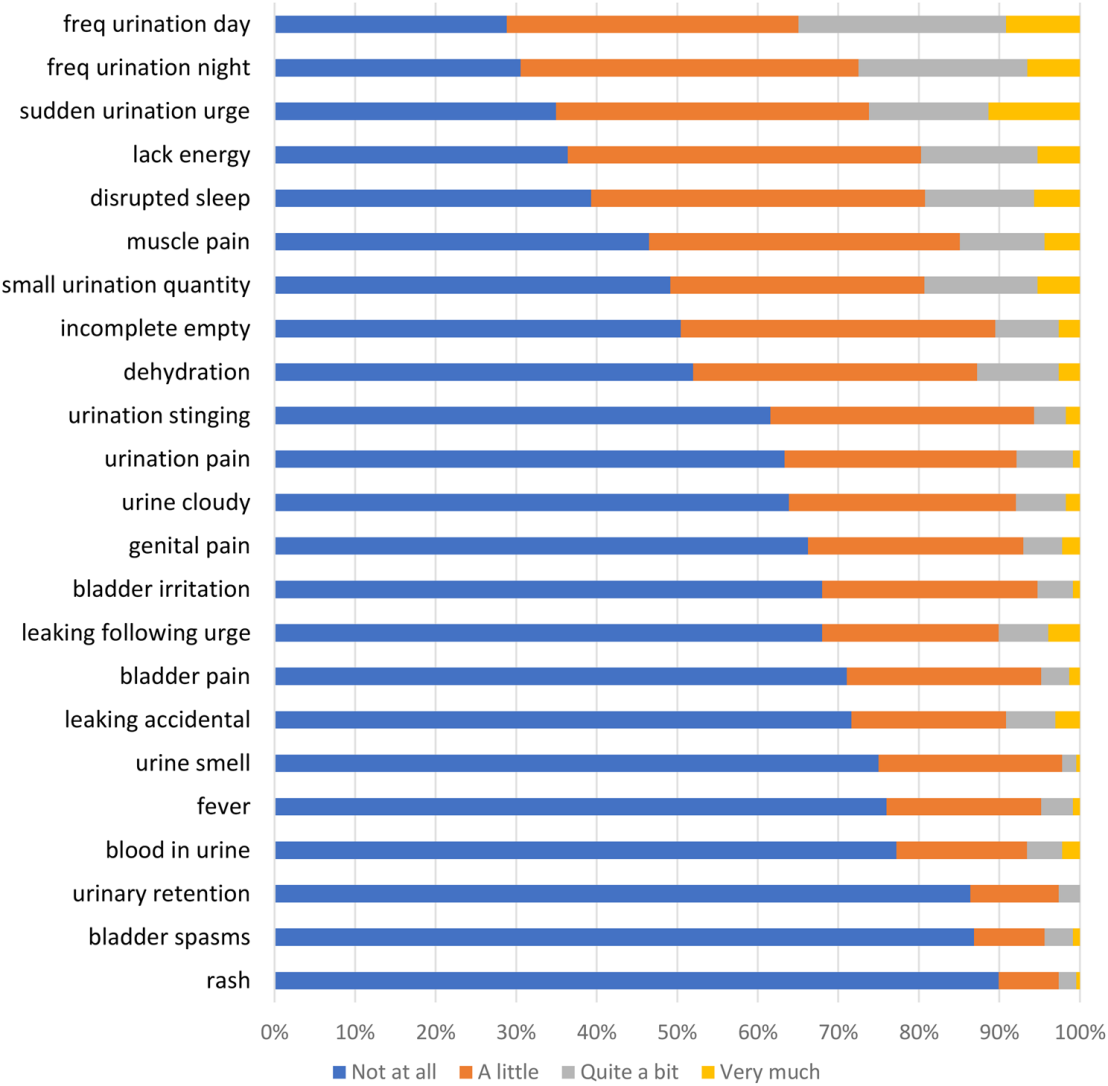
NMIBC-Core 23-item response frequency

**Fig. 3:**
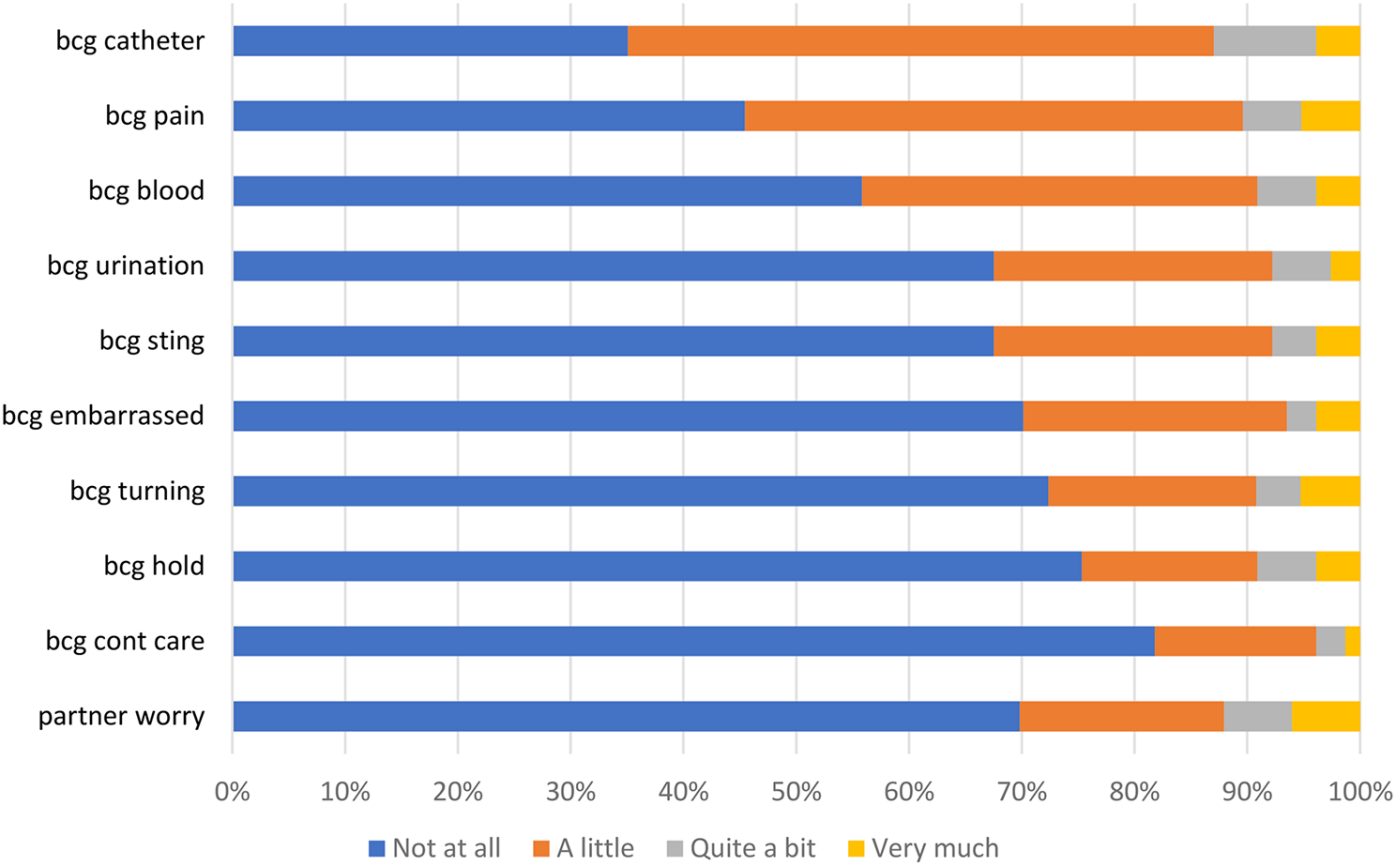
NMIBC-Intra 10-item response frequency

**Fig. 4:**
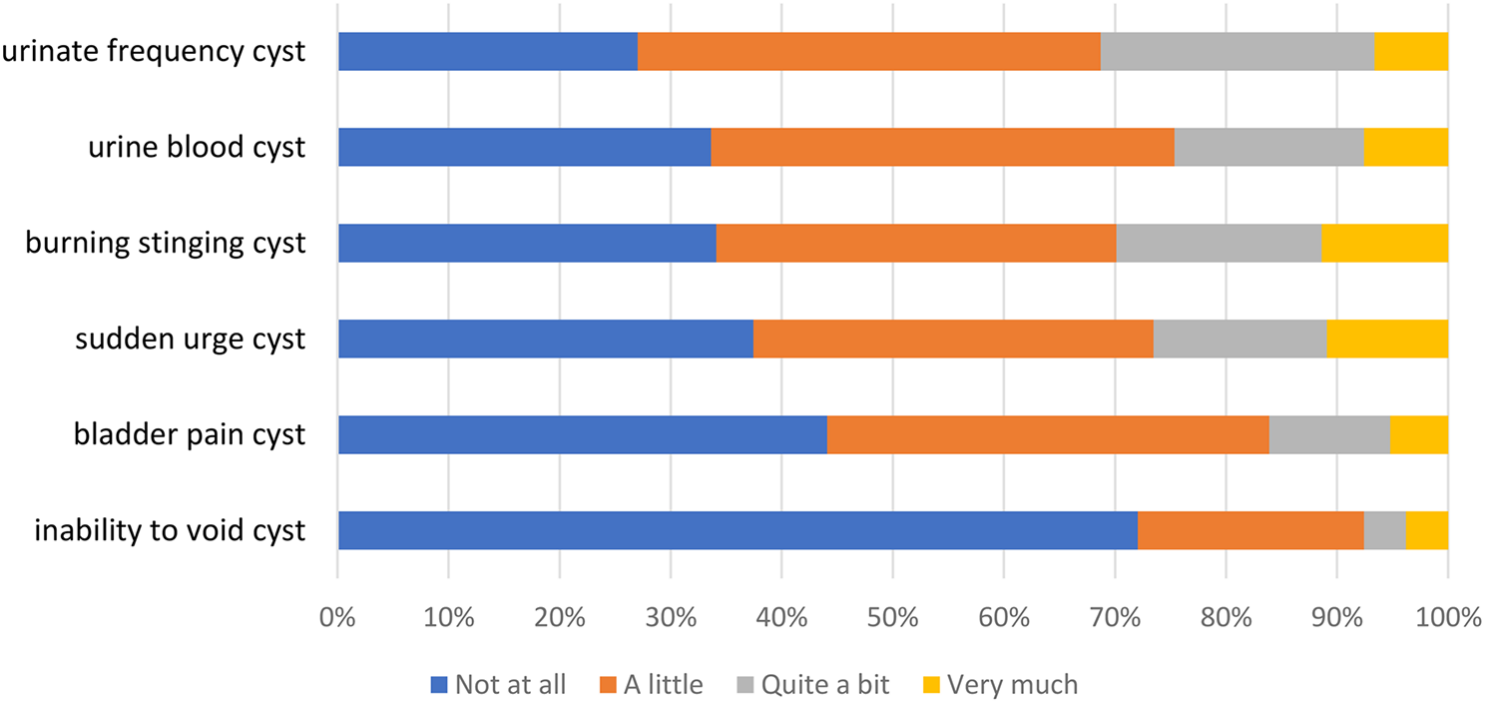
NMIBC-Cyst 6-item response frequency

Item response option frequencies for the three indexes are presented in Figs. 2, 3 and 4. Few participants responded 4 ‘Very much’ to any of the NMIBC-SI items (< 10% of participants responded very much to any of the items), and many participants responded 1 ‘Not at all’ to numerous items. For example, ≥ 50% of participants responded 1 ‘Not at all’ for 16/23 (70%) items in the NMIBC-Core index (Figs. 2) and 8/10 (80%) items in the NMIBC-Intra (Fig. 3). There was greater spread of participants across the response options for the NMIBC-Cyst index (Fig. 4).

All three models tested exhibited good fit according to most fit indices (Table 5). The AIC and BIC both suggested that the one-composite model including both core and cystoscopy index items was best fitting, although the two-composite (core and cystoscopy) model had the highest *R*^2^. Based on these results, we conclude that the core and cystoscopy index items can be represented as separate indexes. A single 29-item index is defensible, although the 6 cystoscopy items are applicable only to patients who have received a recent cystoscopy. For subsequent analyses, we analysed the proximal, cystoscopy, and Intravesical composites separately.

**Table 5:** Fit indices for the three composite models tested

	1 composite (core items only)	2 composites (core and cystoscopy items separate)	1 composite (core and cystoscopy items combined)
*R*^2^	0.262	0.276	0.262
**Fit indices**			
CFI	0.977	0.948	0.980
TLI	0.951	0.860	0.953
RMSEA	0.104	0.065	0.098
SRMR	0.025	0.046	0.024
AIC	-65.1991	-62.5436	-60.4643
BIC	-58.3669	-52.5166	-53.7796

*R*^2^ = variance accounted for; df = degrees of freedom; CFI comparative fit index; TLI Tucker-Lewis Index; RMSEA root mean square error of approximation; SRMR standardised root mean residual. Criteria for good fit were as follows: CFI > 0.9, TLI > 0.9, RMSEA < 0.08, SRMR < 0.05. Akaike and Bayesian Information Criteria (AIC and BIC) calculated to determine the best fitting model

### Index-level performance

The three index summary scores had measures of central tendency close to the low end of the scale range (i.e. predominance of low scores), indicating most participants were not exhibiting many serious difficulties (Table 6).

**Table 6:** Index-level performance

Index (# of items)	Mean	SD	Median	Mode	Possible score range	Observed score range	Floor/Ceiling effect (%)	Skewness
NMIBC-Core (23) *	34.5	8.7	33	35	23–92	23–75	0.4/2.7	1.046
NMIBC-Cyst (6) *^x^	10.7	3.6	10	10	6–24	6–22	0.9/9	0.902
NMIBC-Intra (10) *^x^	15.1	4.6	14	13	10–40	10–34	1.3/6.6	1.851

∗ High scores indicate great bother/impact

^x^ Analysis performed on sub-set of patients who received the treatment and completed treatment-specific modules: cystoscopy, *n* = 211; BCG/Chemotherapy, *n* = 76

Floor effect =% scoring 100 (greatest bother/impact); ceiling effect = % scoring 0 (least bother/impact); *SD* standard deviation

### Reliability

The intraclass correlations for test-retest reliability are shown in Table 7. For the three index scores, test-retest reliability was good (range 0.89–0.91).

**Table 7:** Test-retest reliability for NMIBC-SI indexes

Index	ICC (95% confidence interval)
NMIBC-Core	0.89 (0.84–0.92)
NMIBC-Cyst	0.89 (0.87–0.91)
NMIBC-Intra	0.91 (0.89–0.93)

ICC intraclass correlation coefficient

### Clinical validity

As expected, we found significant differences between no treatment and any treatment groups and between no treatment and chemo/BCG groups for the NMIBC-Core index (Table 8a, b). We also found significant differences between BCG and chemotherapy groups, with significantly higher scores for BCG patients. No other differences between treatment groups were statistically significant, although several others, including all of the NMIBC-Intra comparisons, were moderate to large.

**Table 8a:** Known groups: no treatment vs. treatment (TURBT, chemotherapy, BCG) mean scores

Index	No Tx			TURBT		Chemo		BCG	
	Mean	*n*	Mean		*n*	Mean	*n*	Mean	*n*
NMIBC-Core	33.38	120	37.82		11	36.25	8	37.08	36
NMIBC-Cyst	10.93	42	9.90		10	9.67	3	10.83	6
NMIBC-Intra	17.42	14	14.00		2	12.67	6	14.59	34

BCG Bacillus Calmette-Guerin; TURBT transurethral resection of bladder tumour; Tx treatment

**Table 8b:** Known groups: no treatment vs. treatment (TURBT, chemotherapy, BCG) between group differences

Indexes	Treatment group comparisons	t (*p*-value)	Cohen’s d (95% CI)
NMIBC-Core	No treatment vs. any treatment	2.89 (0.004)	0.44 (0.11, 0.76)
	No treatment vs. chemo/BCG	2.80 (0.006)	0.42 (0.07, 0.78)
	No treatment vs. TURBT	1.32 (0.213)	0.48 (-0.14, 1.11)
	Chemo vs. BCG	0.38 (0.712)	0.13 (-0.66, 0.92)
NMIBC-Cyst	No treatment vs. any treatment	-0.84 (0.406)	-0.21 (-0.77, 0.34)
	No treatment vs. chemo/BCG	-0.47 (0.646)	-0.13 (-0.87, 0.61)
	No treatment vs. TURBT	-0.80 (0.436)	-0.27 (-0.98, 0.43)
	Chemo vs. BCG	0.82 (0.437)	0.44 (-1.25, 2.12)
NMIBC-Intra	No treatment vs. any treatment	-1.56 (0.142)	-0.71 (-1.34, 0.07)
	No treatment vs. chemo/BCG	-1.55 (0.144)	-0.70 (-1.33, 0.06)
	No treatment vs. TURBT	-1.22 (0.295)	-0.48 (-2.11, 1.15)
	Chemo vs. BCG	2.37 (0.033)	0.65 (-0.25, 1.56)

BCG Bacillus Calmette-Guerin; TURBT transurethral resection of bladder tumour

T statistic, p 0.5% significance

## Discussion

This multicentre international study followed the iterative process recommended by the FDA [7] and EORTC [31] for developing PROMs, and tested the conceptual framework of the NMIBC-SI, confirmed it with scoring rules, and assessed reliability and validity of the resultant index. NMIBC-SI was developed using a rigorous, theoretically, and empirically driven process supporting its content and face validity [10, 12, 16]. The CCA results supported all models tested. We conclude that the core and cystoscopy index items can be represented as separate indexes (23 and 6 items respectively), and although our results also support a single 29-item index, the cystoscopy items are applicable only to patients who have received a recent cystoscopy, so it makes more sense to aggregate those items separately.

Item response frequencies illustrate that participants reported few or mild symptoms, perhaps a reflection of > 50% of our field test 2 sample being graded low risk [17], resulting in less variability in responses than expected. The most commonly reported symptoms (daytime/nighttime urinary frequency and urinary urgency) are consistent with what would be expected in this population, with a likely multifactorial etiology, including from the cancer itself, from repetitive procedures on the bladder, from intravesical treatment (e.g. BCG and chemotherapy both elicit bladder irritation, which can manifest as urgency, frequency, bladder irritation, incomplete emptying and small volume voids), and for some, from age-related changes. Although frequent, these symptoms are less serious effects of NMIBC treatment. The next most common group of symptoms (lack of energy/disrupted sleep/small volume voids) could be a direct consequence of frequency/nighttime urinary disruptions. Rash, smell, fever and haematuria, although less commonly reported, are more serious symptoms possibly indicative of infection, and concerning and troublesome to patients, therefore important to measure. In addition, many patients treated for NMIBC can develop recurrent tumors needing future administrations of the same type of therapy. Having a comprehensive assessment of the symptom burden associated with therapy for NMIBC can serve to guide future decisions regarding choice of agent to treat recurrent tumors.

The NMIBC-SI indexes also satisfied psychometric criteria for reliability and validity. Test-retest reliability was strong indicating stability in the construct they were designed to assess, and they were able to discriminate between no treatment and treatment groups providing evidence of clinical utility. Patients who had no treatment experienced lower symptom burden scores compared to treatment group patients. The NMIBC-Intra index was able to discriminate between BCG and chemotherapy groups. BCG patients experienced worse intravesical-specific symptoms compared to chemotherapy patients as expected [32]. 

The NMIBC-SI provides a brief method for clinically relevant assessment of differences in PROs among contemporary treatments for NMIBC, which can provide an evidence base for the ongoing improvement of future therapies for NMIBC. It is intended for self-completion and patients rate the amount of “bother” attributed during the past week on a 4-point response scale. A key requirement for using the NMIBC-SI in clinical trials is having scoring rules yielding index scores sensitive to both improvement (symptom benefit) and deterioration (burden of treatment). The NMIBC-SI indexes are scored by taking the average of each item included in an index with linear rescaling to an observable range of 0-100, with higher scores representing worse symptoms or functional impairment. It is a flexible method where indexes can be selected depending on research aims and included as PRO endpoints in future NMIBC trials to:

- describe baseline burden of disease-defining symptoms (NMIBC-Core);
- assess the extent of symptom benefit or harm with contemporary treatments for NMIBC by prospective assessment of change from baseline in the NMIBC-Core;
- assess specific burden of cystoscopy and/or intravesical therapy (BCG or chemotherapy) by prospective assessment of change from baseline in NMIBC-Cyst and NMIBC-Intra respectively.

NMIBC-SI can also be used in observational studies of patterns and outcomes of care to document common and persistent problems associated with NMIBC treatment, providing information about treatment sequelae for use by both patients and clinicians. Current gaps in evidence limit our understanding of PRO trajectories from diagnosis through to long-term survivorship and treatment effects [10]. 

NMIBC-SI provides an alternative measurement system to existing PROMs, focusing exclusively on proximal disease and treatment-related symptoms, overcoming some of the limitations of existing PROMs previously described [10, 11] Researchers wishing to assess other aspects of HRQL should use the NMIBC-SI together with PROMs developed specifically to assess those HRQL aspects, although care must be taken not to over-burden patients, particularly with questionnaires that overlap in content. The NMIBC-SI is a relatively short symptom-focussed measure that can help clinical researchers meet international directives to “measure what matters” in trials of treatments for NMIBC where symptoms are a key focus [33–35]. 

Our evaluation component had some limitations. Item response frequencies demonstrated lower symptom burden in our study sample than expected, and although the psychometric results were strong, it is uncertain how the instrument would perform in a sample with greater variability. Our inability to test the Intravesical index using CCA due to unexpectedly small numbers of patients experiencing BCG/chemo should be addressed in future research with more targeted recruitment of these patients. Further, clinical validity analysis was also affected by unexpectedly low numbers of respondents in certain clinical groups (patients receiving BCG or chemotherapy at relevant PRO assessment time-points). Further research is needed with a diverse patient sample (e.g. patients experiencing more severe symptoms) to provide additional support for the NMIBC-SI’s measurement properties. A longitudinal observational study is underway that will explore change in PROs over time (acute to 5-year survivorship), the effect of maintenance BCG in addition to induction, the effect of recurrent disease and disease progression over time, and by patient risk category groups [15]. 

In future, the NMIBC-SI could also be used in clinical practice. Although developed to be a PROM for clinical research, its application in clinical practice was also considered during development [36, 37]. The layout, question content, and uniform response format were designed with ease of completion and rapid interpretation in mind. The 23-item NMIBC-SI can be completed within 4-min, and the responses reviewed by clinicians and compared with previous patient assessments within 1-minute if data are captured electronically and auto-generated comparative reports enabled. Implementing this would require resources and infrastructure. Envisaged applications of the NMIBC-SI in clinical practice would be to identify and appreciate the nature, severity, and time course of troublesome symptoms. Ready access to this information should improve shared understanding, discussions, and treatment decisions [33, 38]. 

## Conclusion

Comparative effectiveness research in NMIBC requires a strong evidence-base that incorporates assessment of PROs. Such research is becoming more important in NMIBC due to the rapidly evolving treatment landscape with multiple new therapies being developed and approved. To fully capture and quantify patients’ perspectives, appropriately constructed and validated PROMs are required. The NMIBC-SI provides a brief method for clinically relevant assessment of differences in PROs among contemporary treatments for NMIBC for use in research and potentially clinical practice. The NMIBC-SI would benefit from further examination of its measurement properties in a sample of patients experiencing more severe symptoms.

## Electronic supplementary material

Below is the link to the electronic supplementary material.

Supplementary Material 1

## Data Availability

The datasets generated and analysed during the current study are not publicly available due to follow-up analyses underway, with reports yet to be published, but are available from the corresponding author on reasonable request.

## Declarations

### Clinical trial registration

ClinicalTrials.gov NCT03091764, 27 March 2017. http://clinicaltrials.gov/ct2/showNCT03091764.

## References

[1] Ferlay J, Ervik M, Lam F, Laversanne M, Colombet M, Mery L, et al (2024) Global Cancer Observatory: Cancer Today. https://gco.iarc.who.int/today. Accessed 06 March 2024

[2] Heney NM (1992) Natural history of superficial bladder cancer. Prognostic features and long-term disease course. Urol Clin North Am 19(3):429–433 1636228

[3] Miyake M, Kitamura H, Nishimura N, Miyamoto T, Nakahama T, Fujii T, et al (2024) Validation of non-muscle-invasive bladder cancer risk stratification updated in the 2021 European association of urology guidelines. 5(2):269–280. 10.1002/bco2.305 10.1002/bco2.305 PMC10869660 38371197

[4] Sylvester RJ, Rodríguez O, Hernández V, Turturica D, Bauerová L, Bruins HM et al (2021) European association of urology (EAU) prognostic factor risk groups for Non–muscle-invasive bladder cancer (NMIBC) incorporating the WHO 2004/2016 and WHO 1973 classification systems for grade: An update from the EAU NMIBC guidelines panel. Eur Urol 79(4):480–488. 10.1016/j.eururo.2020.12.033 33419683 10.1016/j.eururo.2020.12.033

[5] Anastasiadis A, de Reijke TM (2012) Best practice in the treatment of nonmuscle invasive bladder cancer. Ther Adv Urol 4(1):13–32. 10.1177/1756287211431976 22295042 10.1177/1756287211431976 PMC3263923

[6] Australian Institute of Health and Welfare (AIHW) (2014) Cancer in Australia: An overview 2014. http://webarchive.nla.gov.au/gov/20150622042034/http://www.aihw.gov.au/cancer/cancer-in-australia-overview-2014/appendixb/. Accessed 22 June 2015

[7] Food and Drug Administration (2009) Patient reported outcome measures: Use in medical product development to support labelling claims. https://www.fda.gov/media/77832/download

[8] Bottomley A, Flechtner H, Efficace F, Vanvoorden V, Coens C, Therasse P, et al (2005) Health related quality of life outcomes in cancer clinical trials. Eur J Cancer 41(12):1697–1709 16043345 10.1016/j.ejca.2005.05.007

[9] Patrick DL, Burke LB, Powers JH, Scott JA, Rock EP, Dawisha S, et al (2007) Patient-reported outcomes to support medical product labeling claims: FDA perspective. Value Health 10(2):S125–137 17995471 10.1111/j.1524-4733.2007.00275.x

[10] Rutherford C, Patel MI, Tait MA, Smith DP, Costa DSJ, Sengupta S, et al (2021) Patient-reported outcomes in non-muscle invasive bladder cancer: A mixed-methods systematic review. Qual Life Res 30(2):345–366. 10.1007/s11136-020-02637-9 32960394 10.1007/s11136-020-02637-9

[11] Rutherford C, Patel MI, Tait MA, Smith DP, Costa DSJ, King MT (2018) Assessment of content validity for patient-reported outcome measures used in patients with non-muscle invasive bladder cancer: A systematic review. Support Care Cancer 26(4):1061–1076. 10.1007/s00520-018-4058-8 29392479 10.1007/s00520-018-4058-8

[12] Rutherford C, Costa DSJ, King MT, Smith DP, Patel MI (2017) A conceptual framework for patient-reported outcomes in non-muscle invasive bladder cancer. Support Care Cancer 25:3095–3102. 10.1007/s00520-017-3717-5 28451912 10.1007/s00520-017-3717-5

[13] Aaronson NK, Ahmedzai S, Bergman B, Bullinger M, Cull A, Duez NJ et al (1993) The European organization for research and treatment of cancer QLQ-C30: A quality-of-life instrument for use in international clinical trials in oncology. J Natl Cancer Inst 85(5):365–376 8433390 10.1093/jnci/85.5.365

[14] Blazeby JM, Hall E, Aaronson NK, Lloyd L, Waters R, Kelly JD, et al (2014) Validation and reliability testing of the EORTC QLQ-NMIBC24 questionnaire module to assess patient-reported outcomes in non-muscle-invasive bladder cancer. Eur Urol 25(14):163–168 10.1016/j.eururo.2014.02.034 PMC4410297 24612661

[15] Rutherford C, King MT, Smith DP, Costa DS, Tait MA, Patel MI (2017) Psychometric evaluation of a patient-reported symptom index for nonmuscle invasive bladder cancer: Field testing protocol. JMIR Res Protoc 6(11):e216. 10.2196/resprot.8761 29117930 10.2196/resprot.8761 PMC5700405

[16] Rutherford C, Tait M-A, Costa D, King M, Smith D, Sengupta S, et al (2020) PD12-08 development of a patient-reported symptom index for use with non-muscle invasive bladder cancer patients using mixed methods. J Urol 203(Supplement 4):e262–e262. 10.1097/JU.0000000000000846.08

[17] Babjuk M, Burger M, Compérat EM, Gontero P, Mostafid AH, Palou J, et al (2019) European association of urology guidelines on non-muscle-invasive bladder cancer (TaT1 and carcinoma in Situ)– 2019 update. Eur Urol 76(5):639–657. 10.1016/j.eururo.2019.08.016 31443960 10.1016/j.eururo.2019.08.016

[18] Wright JG, Feinstein AR (1992) A comparative contrast of clinimetric and psychometric methods for constructing indexes and rating scales. J Clin Epidemiol 45(11):1201–1218 1432001 10.1016/0895-4356(92)90161-f

[19] Feinstein AR (1987) Clinimetrics. Yale University Press, New Haven

[20] Bollen KA, Bauldry S (2011) Three Cs in measurement models: Causal indicators, composite indicators, and covariates. Psychol Methods 16(3):265–284. 10.1037/a0024448 21767021 10.1037/a0024448 PMC3889475

[21] Costa DS (2015) Reflective, causal, and composite indicators of quality of life: A conceptual or an empirical distinction? Qual Life Res 24(9):2057–2065. 10.1007/s11136-015-0954-2 25725599 10.1007/s11136-015-0954-2

[22] Fayers PM, Hand DJ (2002) Causal variables, indicator variables and measurement scales: An example from quality of life. J R Statist Soc A 165(2):233–261

[23] Streiner DL (2003) Being inconsistent about consistency: When coefficient alpha does and doesn’t matter. J Pers Assess 80(3):217–222. 10.1207/s15327752jpa8003_01 12763696 10.1207/S15327752JPA8003_01

[24] Brédart A, Anota A, Young T, Tomaszewski KA, Arraras JI, Melo MDA, H., et al (2018) Phase III study of the European organisation for research and treatment of cancer satisfaction with cancer care core questionnaire (EORTC PATSAT-C33) and specific complementary outpatient module (EORTC OUT-PATSAT7). Eur J Cancer Care (English Lang Edition) 27(1). 10.1111/ecc.12786 10.1111/ecc.12786 29094784

[25] Peipert JD, Beaumont JL, Bode R, Cella D, Garcia SF, Hahn EA (2014) Development and validation of the functional assessment of chronic illness therapy treatment satisfaction (FACIT TS) measures. Qual Life Res 23(3):815–824. 10.1007/s11136-013-0520-8 24062239 10.1007/s11136-013-0520-8

[26] Schuberth F, Henseler J, Dijkstra TK (2018) Confirmatory Compos Anal [Methods] 9. 10.3389/fpsyg.2018.02541

[27] Henseler J (2021) Composite-based structural equation modeling: Analyzing latent and emergent variables. The Guilford Press, New York, NY, US

[28] Costa DSJ, King MT, Henseler J (2024) Modeling forged concepts in patient-reported outcomes using confirmatory composite analysis. [Manuscript in preparation]

[29] Rademaker ME, Schuberth F (2020) cSEM: Composite-Based Structural Equation Modeling. Package version: 0.5.0. https://m-e-rademaker.github.io/cSEM/

[30] Cohen J (1988) Statistical power analysis for the behavioral sciences. L. Erlbaum Associates, Hillsdale, N.J.

[31] EORTC Quality of Life Group (2021) Module Development Guidelines; 5th Edition. Brussels: EORTC

[32] Beeren I, Klerks NE, Aben KK, Oddens JR, Witjes JA, Kiemeney LA et al (2023) Health-related quality of life during the first 4 years after non-muscle-invasive bladder cancer diagnosis: Results of a large multicentre prospective cohort. Eur Urol Oncol. 10.1016/j.euo.2023.11.007 37996278 10.1016/j.euo.2023.11.007

[33] Di Maio M, Basch E, Denis F, Fallowfield LJ, Ganz PA, Howell D et al (2022) The role of patient-reported outcome measures in the continuum of cancer clinical care: ESMO clinical practice guideline. Ann Oncol 33(9):878–892. 10.1016/j.annonc.2022.04.007 35462007 10.1016/j.annonc.2022.04.007

[34] Kamal AH, Bausewein C, Casarett DJ, Currow DC, Dudgeon DJ, Higginson IJ (2020) Standards, guidelines, and quality measures for successful specialty palliative care integration into oncology: Current approaches and future directions. 38(9):987–994. 10.1200/jco.18.02440 10.1200/JCO.18.02440 PMC7082154 32023165

[35] Australian Government (2023) Measuring What Matters Statement. https://treasury.gov.au/publication/p2023-mwm

[36] International Society for Quality of Life Research (prepared by, Aaronson NET, Greenhalgh J, Halyard M, Hess R, Miller D, Reeve B, Santana M, Snyder C) (2015) User’s Guide to Implementing Patient-Reported Outcomes Assessment in Clinical Practice 10.1007/s11136-011-0054-x 22048932

[37] International Society for Quality of Life Research (prepared by Chan E, E. T., Haywood K, Mikles S, Newton L) (2018) Companion Guide to Implementing Patient Reported Outcomes Assessment in Clinical Practice 10.1007/s11136-018-2048-4 PMC8084633 30448911

[38] Pérez-Alfonso KE, Sánchez-Martínez V (2021) Electronic Patient-Reported outcome measures evaluating cancer symptoms: A systematic review. Semin Oncol Nurs 37(2):151145. 10.1016/j.soncn.2021.151145 33773879 10.1016/j.soncn.2021.151145

