# Supplementary material for: Development and psychometric evaluation of a patient-reported symptom index for patients with non-muscle invasive bladder cancer: the NMIBC-SI
Source: J Patient Rep Outcomes. 2025 Mar 27;9:36. doi: 10.1186/s41687-025-00864-7 (PMC11950540; doi:10.1186/s41687-025-00864-7)
Supplement: Supplementary file 1 — Supplementary Material 1 [file 41687_2025_864_MOESM1_ESM.docx]

**NMIBC-SI Online Supplementary materials**

| **Table S1** Field test 1 participant sociodemographic and clinical information (n=220) | |
| --- | --- |
| **Characteristic** | **Total n (%)** |
| **Country region of birth** |  |
| Australia and New Zealand | 106 (48.2) |
| Asia | 11 (5) |
| Middle East and North Africa | 8 (3.6) |
| Northern and Central Europe | 15 (6.8) |
| United Kingdom | 42 (19.1) |
| Southern Europe | 22 (10) |
| Sub-Saharan Africa | 5 (2.3) |
| Americas | 4 (1.8) |
| Missing | 7 (3.2) |
| **Marital Status** |  |
| Single, separated, divorced or widowed | 71(32.3) |
| Married or cohabiting | 146 (66.4) |
| Missing | 3 (1.3) |
| **Living arrangements** |  |
| Live alone | 42 (19.1) |
| Live with partner, identified carer or other | 175 (79.5) |
| Missing | 3 (1.4) |
| **Comorbidity (Charlson Comorbidity Index)*** |  |
| History of Prostate Cancer (0) | 14 (6.4) |
| History of BPH (Benign Prostate Hyperpplasia) (0) | 23 (10.5) |
| Myocardial infarct (+1) | 17 (7.7) |
| Congestive heart failure (+1) | 5 (2.3) |
| Peripheral vascular disease (+1) | 13 (5.9) |
| Cerebrovascular disease (except hemiplegia) (+1) | 8 (3.6) |
| Chronic pulmonary disease (+1) | 10 (4.5) |
| Ulcer disease (+1) | 1 (0.5) |
| Mild liver disease (+1) | 1 (0.5) |
| Diabetes (without complications) (+1) | 23 (10.5) |
| Diabetes with end organ damage (+2) | 2 (0.9) |
| Moderate or severe renal disease (+2) | 8 (3.6) |
| Solid tumor (nonmetastatic) (+2) | 11 (5.0) |
| Leukemia (+2) | 1 (0.5) |
| Lymphoma, Multiple myeloma (+2) | 2 (0.9) |
| Moderate orsevere liver disease (+3) | 2 (0.9) |
| Metastatic solid tumor (+6) | 4 (1.8) |
| No Comorbidities | 118 (53.6) |
| Missing | 27 (12.3) |
| **Charlson Age-Comorbidity Index Scores^1^** |  |
| Mean Comorbidity Score (standard deviation) | 3.2 (1.9) |
| Median | 3 |
| Mode (most commonly occurring score) | 2 |
| Range | 0-10 |
| Missing | 27 (12.3) |
| *Values indicate patients had multiple comorbidities at time of assessment.  ^1^ Higher score indicates greater comorbidity (patients with a score >5 had a 100% risk of dying at one year). Each decade of age ≥50 years is equivalent to a 1-point increase in comorbidity (i.e., 50–59 years=1 point; 60–69 years=2 points). | |

| **Table S2** Field Test 2 participant baseline sociodemographic and clinical information (n=232) | |
| --- | --- |
| **Socio-demographics and clinic-demographics** | **Total n (%)** |
| **Country region of birth** |  |
| Australia & New Zealand | 108 (46.5) |
| Asia | 8 (3.5) |
| Middle East & North Africa | 4 (1.7) |
| Northern & Central Europe | 8 (3.5) |
| United Kingdom | 47 (20.2) |
| Southern Europe | 7 (2.9) |
| Sub-Saharan Africa | 2 (0.8) |
| Americas | 45 (19.4) |
| Missing | 3 (1.3) |
| **Marital status** |  |
| Single, separated, divorced, or widowed | 57 (24.6) |
| Married or Cohabiting | 173 (74.6) |
| Missing | 2 (0.9) |
| **Living arrangement** |  |
| Live alone | 43 (18.5) |
| Live with partner, identified carer, or other | 175 (75.4) |
| Missing | 14 (6) |
